# Giant cystic neck tumor: diagnostic and therapeutic challenges of a rare cutaneous malignancy – a case report

**DOI:** 10.1093/jscr/rjag432

**Published:** 2026-06-13

**Authors:** Sarvesh P Azgaonkar, Gurukeerthi Balakrishna, Avni Agrawal, Anushka Bhushan, Deepak M Nayak, Suresh Pillai

**Affiliations:** Department of Head & Neck Surgery, Kasturba Medical College, Manipal Academy of Higher Education, Manipal, Karnataka 576104, India; Department of Head & Neck Surgery, Kasturba Medical College, Manipal Academy of Higher Education, Manipal, Karnataka 576104, India; Department of Otorhinolaryngology, Kasturba Medical College, Manipal Academy of Higher Education, Manipal, Karnataka 576104, India; Kasturba Medical College, Manipal Academy of Higher Education, Manipal, Karnataka 576104, India; Department of Pathology, Kasturba Medical College, Manipal Academy of Higher Education, Manipal, Karnataka 576104, India; Department of Head & Neck Surgery, Kasturba Medical College, Manipal Academy of Higher Education, Manipal, Karnataka 576104, India

**Keywords:** cystic neck swelling, cystic hygroma, syringocystadenoma papiliferum, cystic metastasis, nevus sebaceous

## Abstract

Syringocystadenocarcinoma papilliferum (SCACP) is a rare cutaneous adnexal neoplasm that may arise from head and neck subsites & often mimic other cystic lesions of neck. We report a case of a giant cystic neck swelling occupying the entire left half of neck posing diagnostic & therapeutic challenges. A nil comorbid man in his 90s presented with a progressive lateral neck swelling for 2 years. An Initial fine needle cytology was non-diagnostic. Magnetic resonance imaging demonstrated extensive involvement by the lesion across both the anterior and posterior triangles of the neck. The tumor was excised by transcervical approach. Final histology with cytokeratin 7,19 & SOX10 positivity suggested SCACP. This case highlights SCACP’s potential for massive, non-infiltrating growth in the neck, distinguishable from other cystic lesions through histology & immunohistochemistry. Successful transcervical excision in a geriatric patient, despite inherent challenges & low tumor aggressiveness reinforces the value of surgical management.

## Introduction

Syringocystadenocarcinoma papilliferum (SCACP) represents an exceedingly rare malignant adnexal neoplasm, classified by World Health Organization as the malignant counterpart to Syringocystadenoma papilliferum (SCAP) [1]. First described in 1980, only 78 cases appear in the English literature, predominantly affecting elderly patients with a slight male predominance and favoring the head and neck region, though may appear in atypical sites like perianal area, chest, suprapubic area, etc. [2] These tumors often arise from preexisting SCAP or nevus sebaceus, originating from apocrine or pluripotent appendageal glands, with histopathological features including papillary projections, nuclear atypia, and mitotic activity distinguishing them from benign SCAP. Despite their rarity, SCACP carries metastatic risk—regional lymph node involvement in 22% and distant spread in 6% of cases—necessitating inclusion in differentials for adnexal malignancies to avoid misdiagnosis as benign lesions. [3] This report is about an unfamiliar and giant cystic mass which involved the neck and was successfully managed by transcervical excision.

## Case report

An elderly man in his early 90s presented with a 2-year history of a slowly progressive left lateral neck swelling. It began as a small bulge and gradually extended to involve the entire left neck and nape. The painless mass caused marked discomfort and restricted neck movement, impairing daily activities, but there were no speech, swallowing, or respiratory symptoms, no ear or nose complaints, and no significant comorbidities. On examination, a 20 × 20 cm soft, cystic, fluctuant, non-tender, bosselated swelling occupied the left neck, extending from the angle of the mandible superiorly, to just above the clavicle inferiorly, up to 2 cm lateral to midline medially, and to the nape posteriorly (Fig. 1). No scars, nevi, dilated vessels, or signs of thoracic inlet obstruction were seen. The swelling was poorly mobile and inseparable from surrounding musculature, but the trachea and larynx were palpable separately. Oral cavity pharynx, larynx, and cranial nerves were normal.

**Figure 1:**
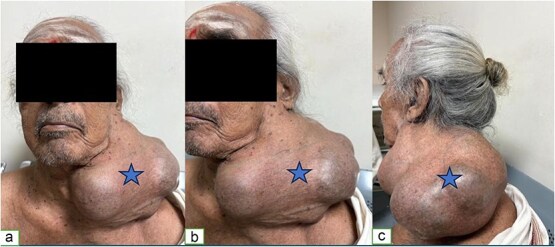
Lateral neck swelling with anterior, posterior, & oblique view. The star indicates the bosselated, stretched & shiny cystic lesion.

### Investigation

The magnetic resonance imaging (MRI) neck of this patient, as illustrated in Fig. 2, showed a large lesion measuring 17 × 13.5 cm on the left side of the neck. The lesion extended posteriorly up to the nape of neck, superiorly abuts the parotid gland and inferiorly extends till the root of the neck. A small portion of the lesion was seen extending between the internal and external carotid artery and the parapharyngeal space on the left side. However, the lesion was lateral to the strap muscles with no extensions into the larynx or pharynx. There were multiple septations and debris seen within this giant swelling with mild T1 hyperintensity. All the above-described features were in favor of a large lymphangioma with the possibility of infection. All other workups were within normal limits, including electrocardiography and 2-D Echo which were done for general anesthesia fitness.

**Figure 2:**
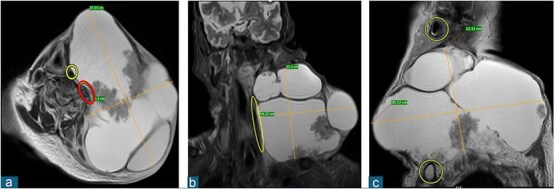
MRI images of the lesion with appropriate sections depicting the extent. (a) Red circle indicates internal jugular vein; yellow circle indicates carotid artery. (b) Yellow circle indicates compressed internal jugular vein. (c) Yellow circles indicate EAC and clavicle.

### Treatment

Given the nonspecific clinical and radiological findings, surgery was performed for debulking and symptomatic relief under nasotracheal intubation. Using an elliptical incision around the midportion of swelling (Fig. 3), subplatysmal flaps were elevated to the mass limits. Dissection medial to the sternocleidomastoid exposed the left common carotid artery, Internal jugular vein and vagus nerve; the adherent, thrombosed left internal jugular vein was sacrificed to improve exposure. The left spinal accessory nerve was sacrificed posteriorly. A rent on the cyst discharged black-straw-colored fluid, facilitating shrinkage and dissection of the postero-inferior margin. The giant lesion was excised en bloc, margins freshened and the skin closed in two layers. A 14-French drain was placed. The patient recovered uneventfully, tolerating oral intake from the evening of surgery. A mild left-sided shoulder weakness was noticed & suggested shoulder physiotherapy. He was mobilized early, with daily drain and vitals monitoring, and discharged on day 5 after drain removal. At 8-month follow-up, he remained free of local recurrence (Fig. 9). Histopathology revealed a 560 g (17 × 13.5 × 6 cm) specimen of malignant dermal adnexal tumor consistent with Syringocystadenocarcinoma papilliferum, featuring cystic components, papillary excrescences, hemorrhage, and purulent content. Microscopy showed thick/thin-walled cysts with branching papillary/villoglandular structures, low-grade nuclei, eosinophilic cytoplasm, apical secretions, rare mitoses, psammoma bodies, and stromal microinvasion, with deep invasion but no lymphovascular or perineural involvement; margins (1 mm) were clear (pT3, pN not assigned; AJCC 8th ed.). IHC was CK7/CK19 positive, ruling out common metastases; CK20/TTF-1/PAX8/AMACR/ERG-1/p63/CEA/GATA3/AR were negative, SOX10 weakly positive (Figs 4–8).

**Figure 3:**
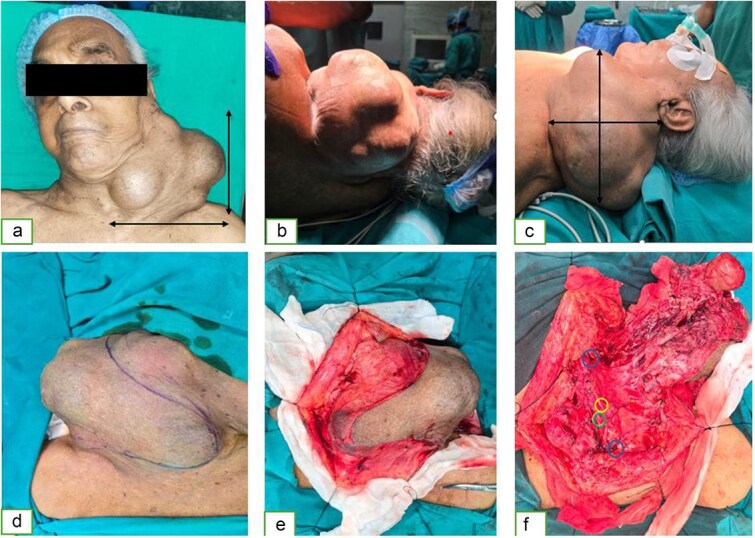
Intraoperative images of the lesion depicting the extent, incision, & relation with vascular structures. (a–c) Gross extent of the lesion. (d) Skin marking over the lesion. (e) Skin flap is raised around the tumor. (f) Relationship of tumor to neurovascular structures of the neck. Blue circles indicate cut ends of IJV; yellow circle indicates vagus nerve; green circle indicates carotid artery.

**Figure 4:**
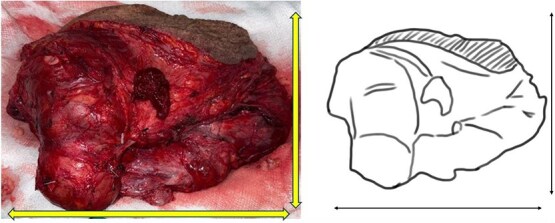
Gross specimen with dimensions. Gross specimen weighed 560 g with exact dimensions of 17 × 13.5 × 6 cm. Additional structures attached – skin ellipse measuring 15.1 × 1.8 cm.

**Figure 5:**
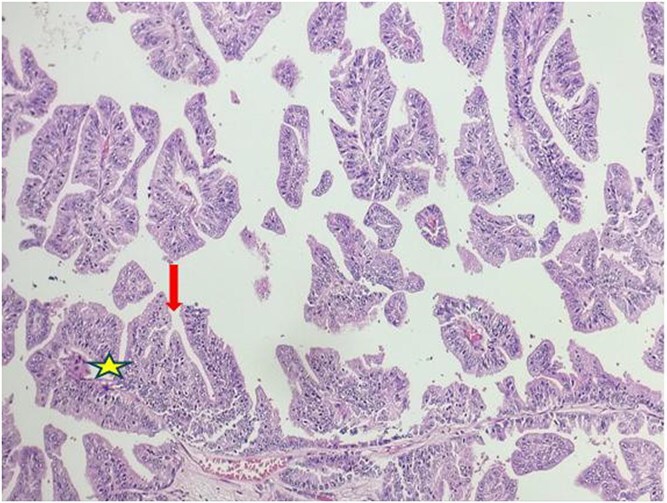
Microscopy showing branching, complex papillary structures with fibrovascular core.

**Figure 6:**
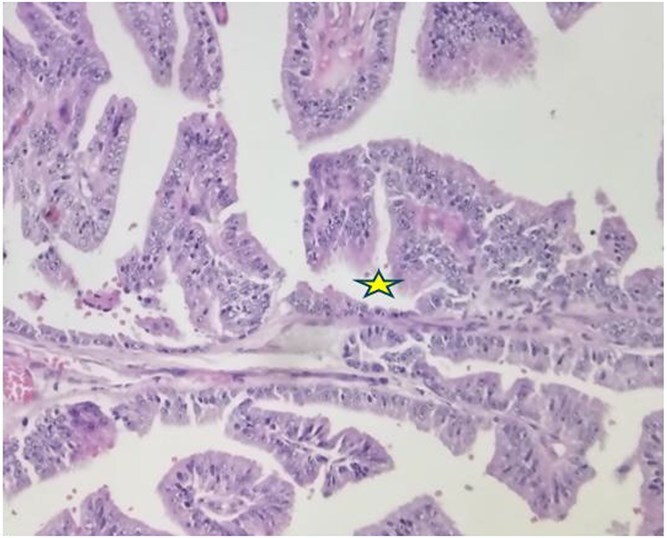
Microscopy showing large areas of superficial epithelium with funnel shaped glandular invagination suggesting dermal origin. The star indicates glandular invagination.

**Figure 7:**
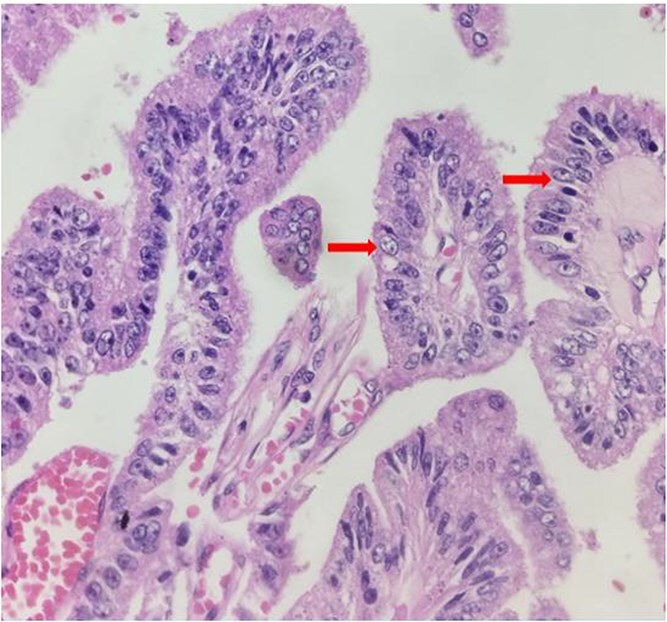
Microscopy showing nuclear atypia and mitotic figures suggestive of malignancy (arrow).

**Figure 8:**
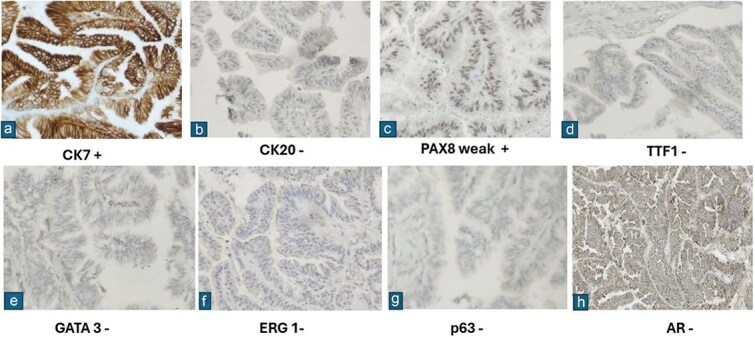
Various results of immunohistochemistry panel.

**Figure 9:**
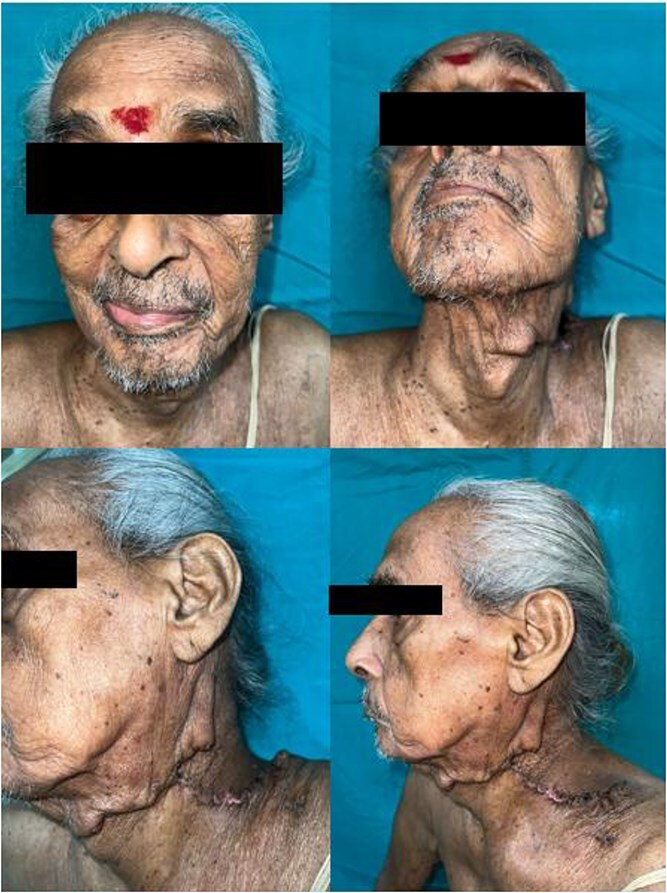
Follow-up images of the patient after complete wound healing.

## Discussion

SCACP is a rare malignant apocrine tumor originating from the pluripotent appendageal glands of the skin. This diagnosis is often preceded by rapid enlargement or ulceration of preexisting nevus or SCAP. This is a tumor so rare that only 78 cases have been reported in the literature. It predominantly affects older men & has a varied presentation such as ulcerated nodule, plaque and rarely non-ulcerative cystic swelling.

A comprehensive literature search including only articles published in English on SCACP of head and neck region using search engines like PubMed and Google scholar identified 44 cases documented till January 2026 (Table 1). Of the reported cases, the median age of presentation was 62.7 years (29–96 years). Majority were males (n = 24;54.5%) & scalp was the most common site, (n = 34/44;75.5%). The neck was one of the less commonly affected sites & reported only in three cases (6.81%). The lease common site among the head and neck region was forehead; only one case has been reported (n = 1/44; 2.27%) The distribution of tumor location within head and neck is shown in Table 2.

**Table 1:** List of published literature on SCACP with demographic details, clinical features, & treatment.

**Author, year**	**Age, y/sex**	**Location**	**Size, cm**	**Duration**	**Clinical features**	**Treatment & outcome**
Dissanayake & Salm, 1980 [3]	74/F	Scalp	6.5 × 5.5 × 3.5	30 years	Enlarging exophytic tumor with copious secretion	Excision No recurrence after 6.75y
Seco Navedo *et al*., 1982 [2]	50/F	Scalp	6.5 × 6.5 × 2.0	since birth	with a recent increase in size	Excision Regional lymph node metastasis
Bondi *et al*. [2] 1996	47/M	Scalp	2.5	Unknown	Solitary ulcerated & crusted	Excision Lost to follow-up
Arai *et al*., 2003 [2]	64/M	Scalp	3.5 × 2.5 × 1.2	2 years	Enlarged erythematous tumor, surrounded by a bloody crust with a macerated white papule	Excision In situ carcinoma
Chi *et al*., 2004 [2]	60/M	Ear (auricle)	4.0 × 4.0 1.0 × 1.0	Since Childhood	Two verrucous plaques, ulcerated with yellow crusts	Mohs micrographic surgery, no recurrence/metastasis after 6 years
Woestenborghs *et al*., 2006 [2]	81/F	Scalp	1.5 × 0.5	Unknown	Raised tumor with bleeding	Excision SCACP in situ with pagetoid spread
Cai *et al*., 2005 [2]	32/F	Neck	1.0	Since birth	Hyperpigmented, slow-growing verrucous nodule	SCACP in situ
Kazakov *et al*., 2010 [2]	56/F	Neck	2.0 × 2.0	10 years	Verrucous ulcerated nodule	Excision SCACP in situ
Kazakov *et al*., 2010 [2]	46/ F	Scalp	3.5	Unknown	Ulcerated, foul-smelling neoplasm	Excision SCACP in situ
Kazakov *et al*., 2010 [2]	67 / M	Scalp	2.5	Unknown	Ulcerated nodule	Excision SCACP in situ
Kazakov *et al*., 2010 [2]	60 / F	Scalp	3.0 × 2.0 × 1.0	30 years	Ulcerated tumor with recent rapid growth	Excision SCACP in situ with pagetoid spread
Kazakov *et al*., 2010 [2]	81 / M	Scalp	2.0	Unknown	Inflammatory plaque	Excision SCACP invasive
Kazakov *et al*., 2010 [2]	58/ M	Forehead	2.5	Unknown	Ruptured cyst	Excision SCACP invasive
Leeborg *et al*., 2010 [2]	86/ F	Neck	4.5 × 4.0 × 4.0	4 months	Large, erythematous to violaceous, asymmetric, exophytic and discolored lesion	Excision Invasive poorly differentiated Local recurrence seen
Aydin *et al*., 2011 [2]	67/ M	scalp	4.0 × 2.0 × 2.0	Since Childhood	Ulcerative nodular lesion	SCACP invasive No Excision
Hoguet *et al*., 2012 [2]	86/ M	Eyelid	0.4	Unknown	Erythematous, ulcerated, curated nodule	Excision SCACP in situ
Bakhshi *et al*., 2012 [2]	45/ F	Scalp	6.0 × 3.0	1 year	Hemispherical swelling with rapid growth, granular surface with erosion and crusts, accompanied by satellite lesions	Excision SCACP invasive with spindle cell variant of a sarcoma
Arslan 2013 [2]	66/F	Scalp	3.0 × 2.2	1 year	Well-defined, erythematosus nodular mass	Excision
Arslan *et al*., 2013 [2]	66/M	Scalp	Unknown	20 years	Multinodular ulcerated lesions	Invasive 3 regional left cervical lymph node metastasis, radical neck dissection, radiation therapy
Peterson *et al*., 2013 [2]	65 / M	Scalp	3.0 × 3.0	Since birth	Flesh-colored, exophytic tumor with serosanguinous exudate, rapid growth for the last 12 months	Excision SCACP invasive
Mohanty *et al*., 2014 [2]	80/F	Scalp	5.0 × 3.3 × 1.3	8 years	Friable, exophytic, tan-pink to red	Excision SCACP in situ
Castillo *et al*., 2014 [2]	32/F	Scalp	2.2 × 1.7 × 1.5	Unknown	Solid and cystic tumor with round, yellowish-white in color and with firm consistency	Excision SCACP in situ Local recurrence
Satter *et al*., 2014 [2]	42/M	Scalp	4.5 × 4.0	1 month	Ulcerated exophytic nodule with satellite papules, easily bled with minor trauma	Excision, posterior neck dissection Lymph node metastasis
Parekh *et al*., 2016 [2]	74/M	Scalp	2.0 × 1.8	Since birth	Enlarging erythematous exophytic nodule with small foci of ulceration	Left neck dissection Lymph node metastasis
Chen *et al*., 2016 [2]	60/F	Scalp	2.8 × 2.0	1 year	Hairless, rough, ill-defined erythematous erosive warty plaque with serosanguinous exudate	Excision SCACP invasive
Zhang *et al*., 2017 [2]	64/ M	Scalp	2.0	1 year	Flat verrucous neoplasm	SCACP in situ, mucinous metaplasia. Multiple distant lymph nodes and lung metastasis (died of disease) Excision + radiotherapy
Zhang *et al*., 2017 [2]	29 F	Scalp	1.5	2 years	Subcutaneous nodule	Excision SCACP in situ
Muthusamy *et al*., 2017 [2]	78/M	Scalp	4.5 × 3.5	10 years	Ulcerated nodular lesion	Excision SCACP invasive
Pagano Boza *et al*., 2019 [2]	63/ M	Eyelid	5.0 × 7.0	7 years	Erythematous nodular lesion with ulceration and induration	Exenteration Invasive SCAP Local recurrence
Alegria Landa *et al*., 2019 [2]	90 / F	Scalp	Unknown	10 months	Eroded nodule with bleeding	SCACP in situ (death 1 year later from an unrelated cause)
McBride *et al*., 2021 [2]	68 / M	Scalp	Unknown	Unknown	Mass with skin erosion	N/A
Kneitz *et al*., 2021 [2]	75 /M	Scalp	2.5 × 1.8	Since birth	Nodular mass (growth in 6 months preceding diagnosis)	Excision SCACP invasive
Zilberg *et al*., 2022 [2]	77/M	Scalp	0.3	3 months	Pearly papule	SCACP invasive Mohs micrographic surgery
Cornejo *et al*., 2024 [2]	71/M	Scalp	Unknown	Unknown	Unknown	SCACP invasive
Cornejo *et al*., 2024 [2]	57/M	Scalp	Unknown	Unknown	Unknown	SCACP in situ
Cornejo *et al*., 2024 [2]	93/M	EAR	Unknown	Unknown	Unknown	SCACP invasive
Cornejo *et al*., 2024 [2]	66/ M	Scalp	Unknown	Unknown	Unknown	SCACP in situ
Cornejo *et al*., 2024 [2]	63/ F	Scalp	Unknown	Unknown	Unknown	SCACP invasive
Cornejo *et al*., 2024 [2]	29 / F	Scalp	Unknown	Unknown	Unknown	SCACP invasive
Cornejo *et al*., 2024 [2]	64 /F	Scalp	Unknown	Unknown	Unknown	SCACP in situ
Cornejo *et al*., 2024 [2]	96/M	EAR	Unknown	Unknown	Unknown	SCACP invasive
Dupont *et al*., 2023 [2]	74 / F	Scalp	Unknown	Since childhood	Slow-growing tumor	SCACP invasive Recurrence on the left conjunctiva Platinum salts and 5-Fluorouracil chemotherapy for 6 months, followed by 3 months chemoradiotherapy
Bashinskaya *et al*., 2023 [2]	83 / M	Scalp	1.6 × 1.6	Several months	Erythematous and tender nodule with hyperkeratotic scale	SCACP invasive Mohs micrographic surgery
Verma *et al*., 2022 [2]	40 / F	Ear (external auditory canal)	16.1 × 9.4 × 7.6	1.5 years	Polypoidal firm mass with watery discharge	Excision SCACP invasive
Present case, 2026	94/M	Neck	17 × 13.5 × 6	2 years	Non-ulcerative massive cystic swelling	Transcervical excision SCACP invasive Follow up −8 months no recurrence

**Table 2:** Based on the available literature, distribution of SCACP in head and neck region.

**Location**	**No. of cases**	**Percentage (%)**
Scalp	34	75.5
Ear	4	8.8
Neck	4	8.8
Eyelid	2	4.4
Forehead	1	2.2

Among the 44 cases, ulceration was the most common presenting symptom (n = 20/44; 45.54%) The tumor size details were available for 32 cases, with an average diameter of 2.85 cm. The duration of presenting symptoms was reported in 27 cases; of these 18 reports provided specific duration. Overall average duration of all these patients was 98.6 months. Interestingly three patients had this tumor since childhood & five of them had since birth.

However, our case is unique in view of unusual presentation i.e. mimicking cystic metastasis & less common site i.e. neck. Despite diagnostic challenges & advanced age our case demonstrates that biological age assessed by G8 geriatric screening tool is a more factor in determining surgical suitability than chronological age, allowing for aggressive management that is both safe and effective. Prompt medical intervention, including imaging and excisional biopsy, remains crucial for a definitive diagnosis. Until now, there are no reliable clinical or histological indicators to determine the prognosis. Further studies on many cases with complete follow-up data are still needed to draw definitive conclusions.
